# Biomarkers for an early diagnosis of immune effector cell associated-hemophagocytic syndrome

**DOI:** 10.3389/fimmu.2025.1635062

**Published:** 2025-08-14

**Authors:** Edoardo Campodonico, Piera Angelillo, Matteo Doglio, Alessia Ugolini, Maria Teresa Lupo Stanghellini, Maddalena Noviello, Elena Tassi, Federica Pedica, Gregorio Maria Bergonzi, Lorenzo Lazzari, Alessandro Bruno, Elisa Diral, Maurilio Ponzoni, Consuelo Corti, Jacopo Peccatori, Annalisa Ruggeri, Andrés José Marìa Ferreri, Chiara Bonini, Fabio Ciceri, Matteo G. Carrabba, Raffaella Greco

**Affiliations:** ^1^ Hematology and Bone Marrow Transplantation Unit, IRCCS San Raffaele Scientific Institute, Milan, Italy; ^2^ University Vita-Salute San Raffaele, Milan, Italy; ^3^ Experimental Hematology Unit, Division of Immunology, Transplantation and Infectious Diseases, IRCCS San Raffaele Scientific Institute, Milan, Italy; ^4^ Cell Therapy Immunomonitoring Laboratory (MITiCi), Division of Immunology, Transplantation and Infectious Diseases, IRCCS San Raffaele Scientific Institute, Milan, Italy; ^5^ Pathology Unit, IRCCS San Raffaele Scientific Institute, Milan, Italy; ^6^ Oncologia ed Ematologia Oncologica, Azienda USL della Valle d’Aosta, Aosta, Italy

**Keywords:** CAR (chimeric antigen receptor) T cells, B cell lymphoma, HLH - hemophagocytic lymphohistiocytosis, biomarkers, hyperinflammation, IEC-HS

## Abstract

Hemophagocytic lymphohistiocytosis (HLH) is a hyper-inflammatory syndrome characterized by deficient NK-cell activity, cytokine storm and altered T-cell immunity, potentially sustained by multiple triggers. Recently, a hyperinflammatory condition resembling HLH has emerged as a potential complication of CAR T-cells. HLH represents a diagnostic conundrum due to its rarity, non-specific presentation and lack of validated biomarkers. We investigated a panel of serum cytokines which represent candidate markers to diagnose HLH after CAR-T. We analyzed 2 patients affected by B-cell lymphomas who received anti-CD19 CAR-T and developed HLH defined according to multiple diagnostic criteria. We identified four controls who did not develop HLH: one with CRS without cytopenia, one with cytopenia with CRS, one with both and one with none. We selected a set of acute-phase molecules including CD163, IL33R (quantitated through ELISA), CTLA-4, and CD80 (quantitated through Luminex) and studied their trend in frozen sera collected at parallel time-points. We studied case sera and compared them with controls. All patients developing CRS showed an early peak in IL2R, CD80, CTLA-4 and IL33R, followed by a second, more marked increase in case of HLH, absent in controls. The kinetics of the selected markers suggests that timing and extent of changes might help discriminating HLH from other causes of fever or cytopenia. Serum CD163, CTLA-4, CD80 and IL33R deserve prospective assessment as promising biomarkers to assist in the differential diagnosis between CRS, HLH and non-inflammatory cytopenias after CAR-T.

Chimeric antigen receptor T (CAR T)-cells have brought about a paradigm shift in the treatment of several lymphoid and plasma cell malignancies (1). This plethora of cell therapies is accompanied by peculiar toxicities, partly related to the cytokine storm induced by T-cell infusion, exposing patients to non-negligible morbidity and potentially jeopardizing treatment outcomes. Cytokine release syndrome (CRS), immune-effector cell associated neurotoxicity syndrome (ICANS) and immune-effector cell associated hematotoxicity (ICAHT) are the most relevant and widely studied (2, 3). In addition, secondary hemophagocytic lymphohistiocytosis (HLH), a hyperinflammatory syndrome which has already been extensively described in multiple autoinflammatory, infectious or neoplastic conditions, is garnering significant attention in this field (4). HLH is characterized by variable degrees of cytopenias, fever, hepatosplenomegaly, elevated inflammatory markers, histological signs of hemophagocytosis and defective NK cell function. Classification criteria commonly used for HLH have been issued in 2007 by the Histiocyte society (HLH-2004) and in 2014 by a French cooperative group (HScore) (5, 6).

The mechanisms underlying CAR T-cell associated HLH remain poorly elucidated, and the presence of concomitant confounding factors (CRS, multi-factorial cytopenia) make its diagnosis elusive. Both the American Society for Transplant and Cell Therapy (ASTCT) and the European Bone Marrow Transplantation group (EBMT) have started cooperative efforts to characterize this disorder (3, 7, 8). So far, two provisional entities have been distinguished based on the time of onset and concomitant CRS/ICANS. The former, CRS/macrophage activation syndrome (CRS/MAS), is an early hyperinflammation with many features of HLH occurring soon after cell infusion that blends with classical CRS and might simply represent a subset of severe CRS. The latter, immune effector cell associated-hemophagocytic syndrome (IEC-HS) begins slightly later, after a period of apparent recovery from initial inflammatory complications, if at all present. However, differential diagnosis of severe CRS from IEC-HS is particularly challenging, due to poor specificity of existing criteria and significant overlap among inflammatory complications. In this respect, the ASTCT has recently proposed new classification criteria to define IEC-HS and discriminate it from other, more frequent, inflammatory complications (3, 8). Moreover, the lack of a standardized nomenclature generates confusion for clinicians tasked to diagnose such conditions and exposes them to the risks of both over- and undertreatment. Cytokine profiling in patients experiencing HLH after CD22 CAR T-cell infusion reveals a secondary peak of IFNγ, MIP-1α, and IL-8, but IL-6 remains low. In this context, the validation of new biomarkers to assist differential diagnosis is an urgent unmet medical need (9).

In our center, we reported 2 patients who received anti-CD19 CAR-T for B-cell lymphoma and developed IEC-HS. Based on literature data demonstrating their role in NK and T-cell mediated inflammation, we investigated a panel of serum markers as candidate adjuvants to diagnose CRS/MAS and IEC-HS, including soluble CD163, IL33R, CTLA-4, and CD80, and studied their trend in frozen sera collected at sequential time-points, before and after infusion. As control, we assessed CD25 levels (IL2R), a well-known HLH marker (5, 10, 11). We compared these 2 patients with 4 unaffected controls (see **Table 1** for detailed clinical information).

**Table 1:** Summary of patient characteristics.

Upn	Gender	Age	Diagnosis	Prior treatment lines	Product	CRS	ICANS	Treatment	HLH	Onset	HLH-treatment	Peak Ferritin (ng/mL)	Prolonged cytopenia (3 mo.)	Treatment (cytopenia)	Outcome
1	M	58	MCL	R-CHOP, HDT+ASCT, Ibrutinib, AlloHCT	Brexu-ce	G1	n..	Tocilizumab (I); DEX;	Yes	D11	MPD, CsA, Anakinr	6929	No	n..	HLH resolved; disease relapse at 4 mo. CR after salvag
2	M	65	DLBCL, NOS	R-CHOP, HDT+ASCT	Axi-ce	G3	G3	Tocilizumab (II); DEX;	Yes	D21	MPD, CsA, Anakinra, VP16	5589	n..	n..	Death due to HLH
3	M	41	PMBCL	R-DA-EPOCH; HDT	Axi-ce	G1	n..	Tocilizumab (III); DEX;	No	n..	n..	226	No	n..	Alive in CR
4	F	69	DLBCL, NOS	R-CHOP/MATRix, HDT+ASCT;	Axi-ce	G1.	n..	Tocilizumab (I)	No	n..	n..	345	G3 neutropenia, G3 anemia, G4 thrombocytopeni	n..	Prolonged cytopenia in FU
5	F	70	DHL	R-CHOP; MATRix	Axi-ce	G1	G2	Tocilizumab (I); DEX;	No	n..	n..	2121	G4 neutropenia, G3 anemia, G4 thrombocytopeni	Autologous CD34+ boost	Alive in CR
6	M	52	HGBCL, NOS	R-CODOX-M-IVAC; HDT+ASCT	Tisa-ce	n..	n..	n..	No	n..	n..	625	No	n..	Alive in CR

AlloHSCT, allogeneic hematopoietic stem cell transplantation; ASCT, autologous stem cell transplantation; CR, complete remission; CRS, cytokine release syndrome; CsA, cyclosporin A; DLBCL, NOS, diffuse-large B cell lymphoma, not otherwise specified; DHL, double-hit lymphoma; FU, follow-up; HLH, hemophagocytic lymphohistiocytosis; HDT, high-dose chemotherapy; HGBCL, NOS, High-grade B cell lymphoma, not otherwise specified; ICANS, immune-effector cell associated cytotoxicity; MCL, mantle cell lymphoma; MPD, methylprednisolone; PMBCL, primary mediastinal B cell lymphoma; UPN, unified patient number; VP16, etoposide.

The first patient was a heavily pre-treated 58-year-old male affected by mantle cell lymphoma (MCL) who underwent brexucabtagene autoleucel (brexu-cel) for a relapse post-allogeneic stem cell transplant. After a G1 CRS on D+4, treated with a single tocilizumab dose, he developed a second febrile episode on D+11 with concomitant alteration in hepatic enzymes and hyperbilirubinemia, initially interpreted as a potential graft-versus-host disease (GvHD) flare and treated with steroids. Due to worsening cytopenia, hypofibrinogenemia, and liver and bone marrow bopsies consistent with hemophagocytosis (**Supplementary Figure 1**), fulfilling six of the HLH-2004 criteria, all major ASTCT criteria as well as 5 minor ones, we added anakinra and cyclosporin on day +25. HScore at that time was 301, indicating a very high probability (>99%) of IEC-HS; sIL2R/Ferritin ratio was 0,67. Subsequently, he underwent full recovery and PET-scan documented complete remission at D+90.

The second patient was a 65-year-old male affected by refractory diffuse large B cell lymphoma (DLBCL) who received axicabtagene ciloleucel (axi-cel) and was admitted to the ICU on day +6 for a G3 CRS with concomitant G2 ICANS treated with inotropes, non-invasive ventilation, dexamethasone and broad-spectrum antibiotics due to severe neutropenia. Upon corticosteroid tapering, he developed pancytopenia, hepatocellular damage and inflammatory markers consistent with IEC-HS (elevated IL2R, triglycerides, ferritin). HLH diagnosis was made on day +21 when all major ASTCT criteria were fulfilled. The HScore was 152, indicating a 52% probability of IEC-HS, whereas sIL2R/Ferritin ratio was 0,55. Unfortunately, the patient had a poor response to high-dose steroids and anakinra and ultimately succumbed to multi-organ failure on day +42.

As controls, we identified four CAR T-cell recipients: one who developed CRS without significant cytopenia, one who developed long-term cytopenia without prior inflammatory issues, one with both and one with none of them. Three of the controls received axi-cel and one tisagenlecleucel (tisa-cel).

Samples were collected and banked in the context of the OSR CAR-T observational protocol. Time-points were pre-determined and included pre-lymphodepletion, infusion, D+3, D+14, D+21, D+28 and monthly thereafter. We analyzed soluble IL2R, CD163, CD80, CTLA4 and IL33R at each available time-point.

We assessed the serum concentration of our investigational markers by ELISA (see **Figure 1**). Soluble IL33R and CD163 are macrophage associated markers, whose levels correlate with the inflammatory state and macrophage activation. Soluble IL33R is a decoy receptor that scavenges IL33, preventing its stimulating effect on Tregs, thus exerting a pro-inflammatory role, while sCD163 is reported to have an inhibitory effect on T lymphocytes. Both these markers show preferential increase in patients with secondary HLH compared to healthy subjects and those with primary HLH (10, 11). However, their dynamics in HLH post-CAR-T cells has never been explored.

**Figure 1:**
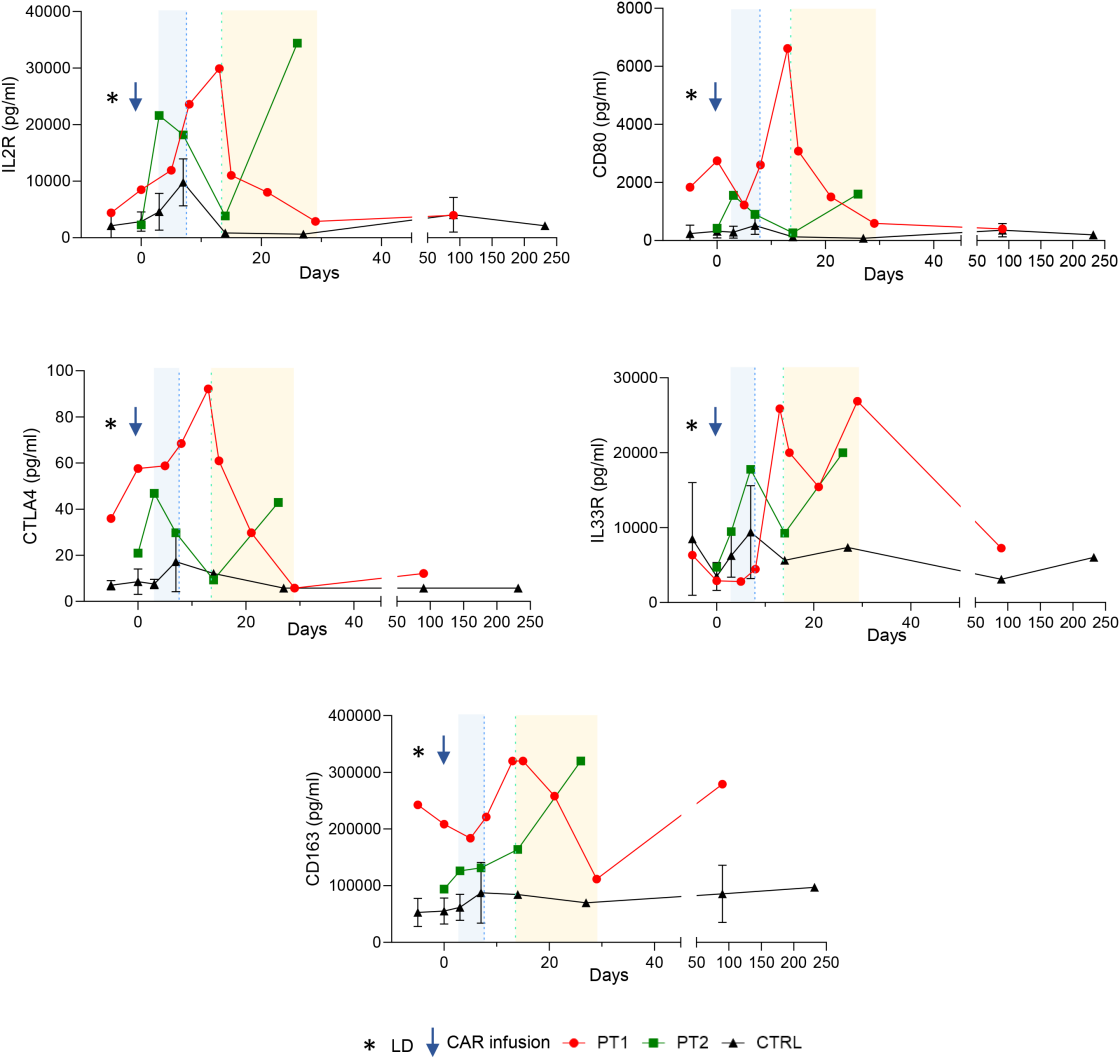
Soluble IL2R, CTLA4, CD163, CD80 and IL33R trends for patients who did (red dots, green squares) and did not develop HLH (black triangles, plotted as aggregated data for simplicity) at parallel time-points. Blue band represents CRS clinical manifestations, whereas yellow band represents HLH. Patient 1 (MCL, Brexu-cel) and patient 2 (DLBCL, Axi-cel) showed remarkably higher serum levels than aggregated controls. LD, lymphodepletion.

We decided to investigate the value of soluble CD80 and CTLA4 and their relationship with post-CAR-T cell HLH. Both these molecules are T-cell regulators. Soluble CD80 is release by monocytes and B cells, whereas sCTLA-4 is released by different cell types, including regulatory T cells and monocytes. Both molecules can exert alternate stimulatory or inhibitory functions on T cells (12, 13). Their role in the context of post-CAR-T cell HLH has never been explored.

Compared to the control group, patient 1 showed similar baseline levels of sIL33R and increased levels of sCTLA-4, sCD163 and sCD80 before cell infusion. Two weeks after treatment, at HLH onset, we observed a marked increase of all the evaluated markers compared to the controls, concomitantly with the appearance of the second febrile episode and the worsening clinical conditions. Subsequently, sCD80, sCD163 and sCTLA-4 plummeted, in line with the clinical improvement, whereas sIL33R displayed an initial reduction, followed by a second peak at day +28. As expected, sIL2R peaked 2 weeks after CAR-T cell infusion, compatible with the diagnosis of IEC-HS, followed by a gradual decrease in line with the clinical improvement. At day +100, after HLH resolution, the level of all the markers except sCD163 was comparable with that of the control group.

Patient 2 showed a rapid increase in sIL2R, sCD80, sCTLA-4 and sIL33R immediately after CAR-T cell infusion, compared to the control group. The examined markers underwent a gradual decrease at subsequent timepoints, reaching levels comparable with that of the control group by day 21. Later on, in line with further clinical deterioration, they underwent a second peak (D+28). Conversely, sCD163 showed a slower but progressive increase, reaching the peak of concentration 4 weeks after CAR-T cell infusion.

Unfortunately, no data were available concerning CAR T-cell kinetics in peripheral blood or in tissue biopsies at the time.

In conclusion, CRS/MAS and IEC-HS are elusive entities, requiring further clinical and translational investigations. Prompt diagnosis and treatment are hampered by the difficulties in ruling out differentials. We investigated a panel of candidate markers which might represent plausible co-adjuvants in the distinction between HLH and other CAR-associated hyperinflammatory syndromes. Soluble CD163, CTLA4, CD80 and IL33R showed a more marked increase in patients with CRS/MAS and IEC-HS compared to CRS alone or negative controls, thus representing promising biomarkers to distinguish CRS/MAS and IEC-HS from CRS and non-inflammatory new-onset cytopenia. So far, these markers chiefly reflect the severity of inflammation and shall be not used as diagnostic tools. However, we believe that broader prospective studies are warranted to study their kinetics across timepoints and potentially validate their role to discriminate distinct clinical events such as CRS and IEC-HS at relevant timepoints.

## Data Availability

The original contributions presented in the study are included in the article/**Supplementary Material**. Further inquiries can be directed to the corresponding authors.

## Supplementary material

Supplementary Figure 1 Liver biopsy samples collected from patient 1 via transjugular biopsy. **(A)** Hematoxylin-eosin staining, 10x: portal tract with inflammatory population damaging the biliary epithelium and associated to oedema; **(B)** Hematoxylin-eosin staining, 20x: severe canalicular and hepatocellular cholestasis; **(C)** Hematoxylin-eosin staining, 20x: biliary infarct; **(D)** Immunohistochemistry, CK7 antibody, 20x: irregular shape of the bile duct with mild ductular proliferation; **(E)** Immunohistochemistry, CD3 antibody 20x: numerous T lymphocytes in the portal tract conditioning the damage of bile duct epithelium; **(F)** Hematoxylin-eosin staining, 20x: hemophagocytosis.

Supplementary Figure 2 panel displaying trends in common biomarkers of IEC-HS (and HLH in general) for patient 1 (red circles) and patient 2 (green squares) over time after CAR T-cell product infusion. **A**: AST, **B**: CRP, **C**: ANC; **D**: ALT; **E**: ferritin; **F**: Hemoglobin; **G**: Triglycerides; **H**: LDH; **I**: Platelets.
