# Supplementary figures and images for: Biomarkers for an early diagnosis of immune effector cell associated-hemophagocytic syndrome
Source: Front Immunol. 2025 Aug 14;16:1635062. doi: 10.3389/fimmu.2025.1635062 (PMC12390792; doi:10.3389/fimmu.2025.1635062)

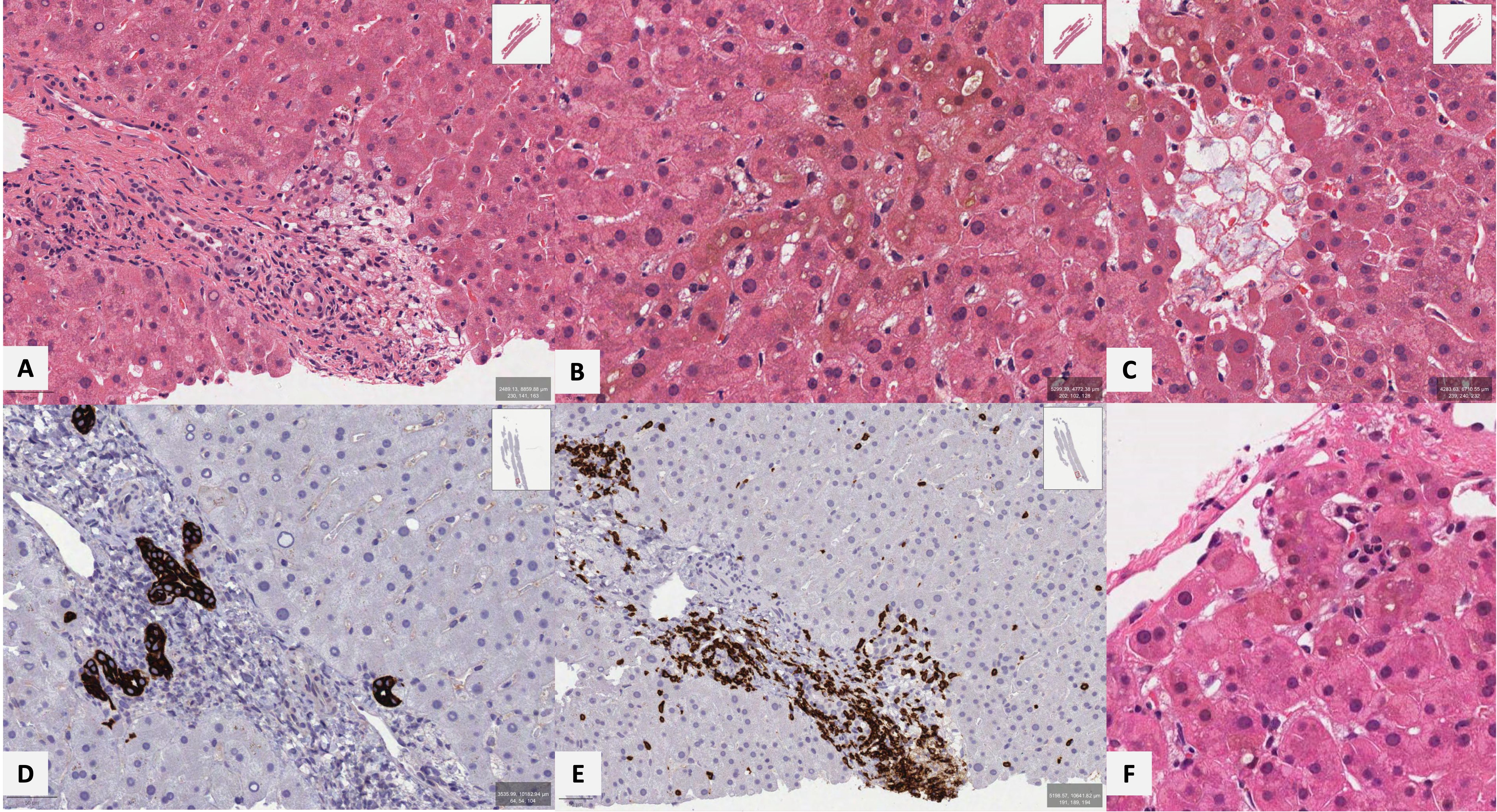

Supplement: Supplementary Figure 1 — Liver biopsy samples collected from patient 1 via transjugular biopsy. (A) Hematoxylin-eosin staining, 10x: portal tract with inflammatory population damaging the biliary epithelium and associated to oedema; (B) Hematoxylin-eosin staining, 20x: severe canalicular and hepatocellular cholestasis; (C) Hematoxylin-eosin staining, 20x: biliary infarct; (D) Immunohistochemistry, CK7 antibody, 20x: irregular shape of the bile duct with mild ductular proliferation; (E) Immunohistochemistry, CD3 antibody 20x: numerous T lymphocytes in the portal tract conditioning the damage of bile duct epithelium; (F) Hematoxylin-eosin staining, 20x: hemophagocytosis. [file Image1.jpeg]

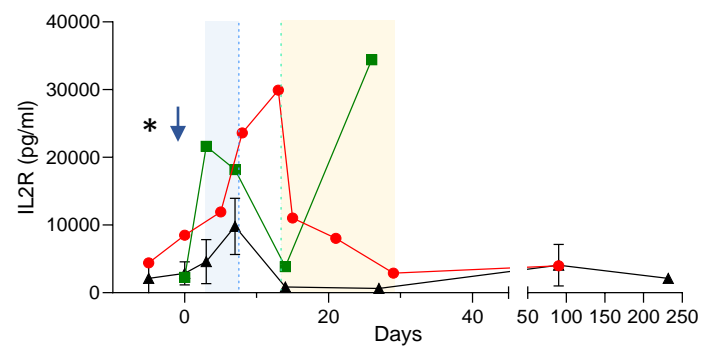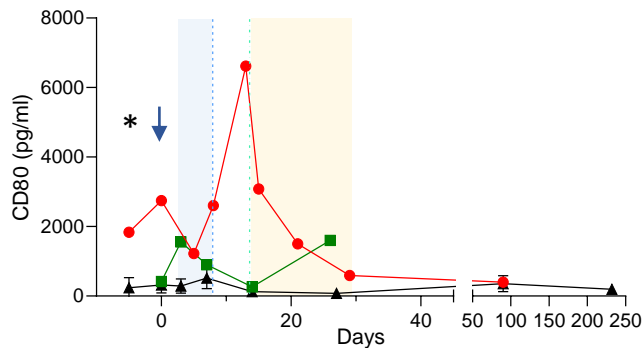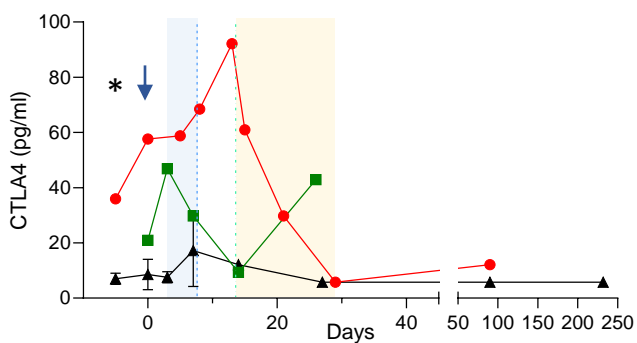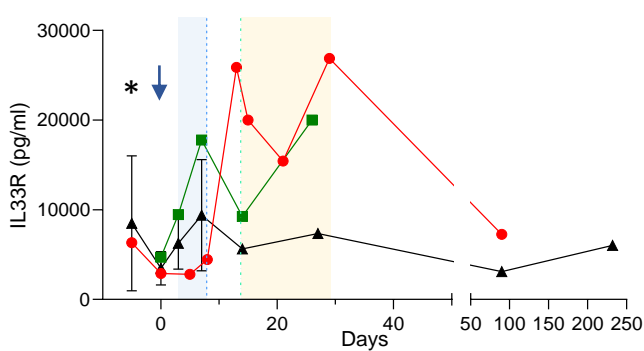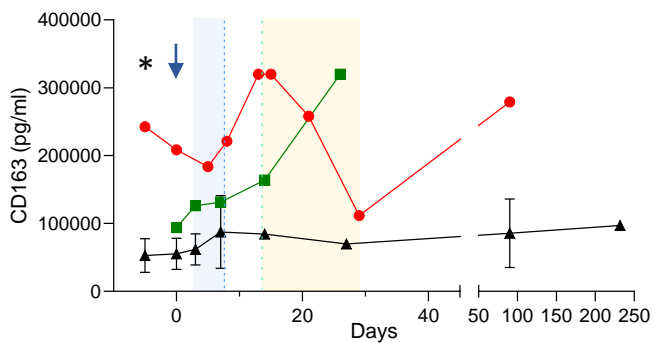

\* LD ↓ CAR infusion —●— PT1 —■— PT2 —▲— CTRL

Supplement: Supplementary Figure 2 — panel displaying trends in common biomarkers of IEC-HS (and HLH in general) for patient 1 (red circles) and patient 2 (green squares) over time after CAR T-cell product infusion. A: AST, B: CRP, C: ANC; D: ALT; E: ferritin; F: Hemoglobin; G: Triglycerides; H: LDH; I: Platelets. [file DataSheet1.pdf]

**a**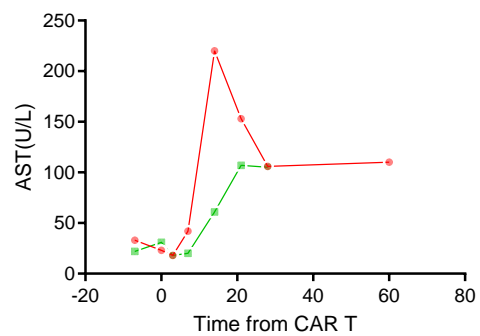**b**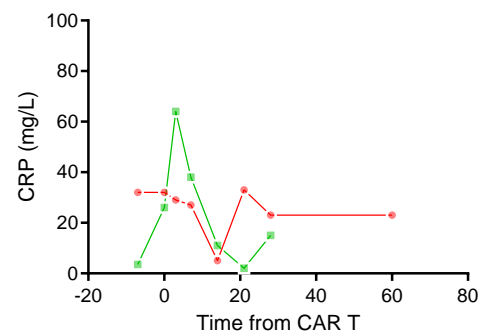**c**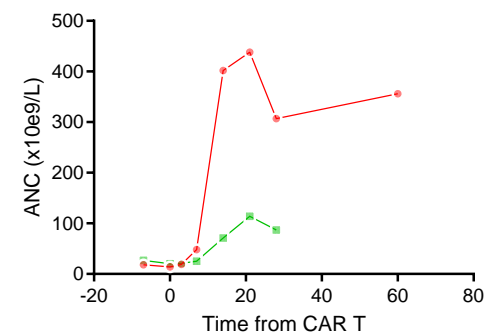**d**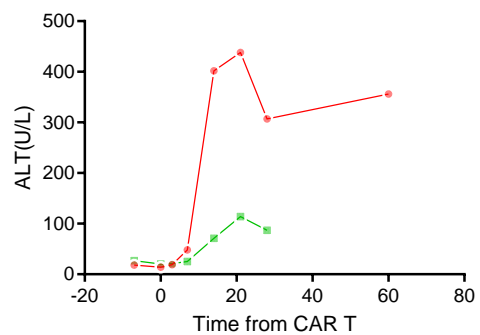**e**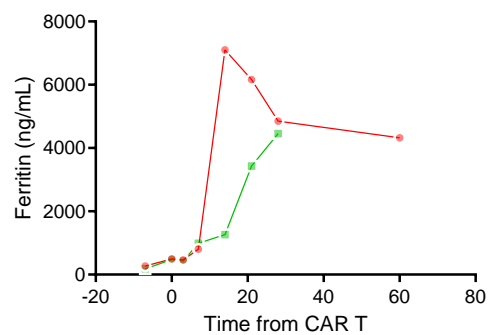**f**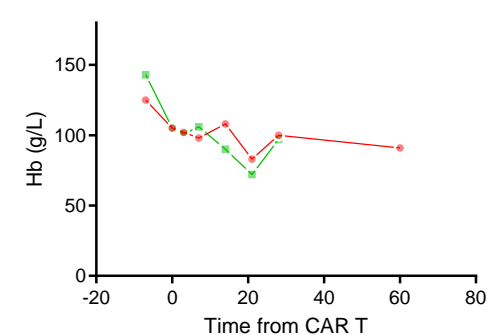**g**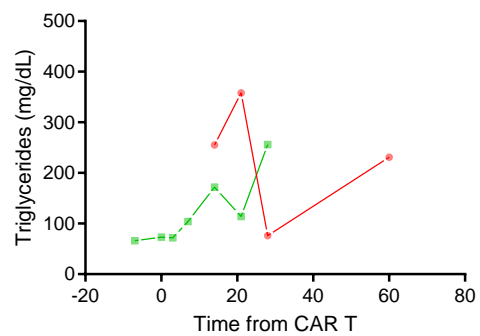**h**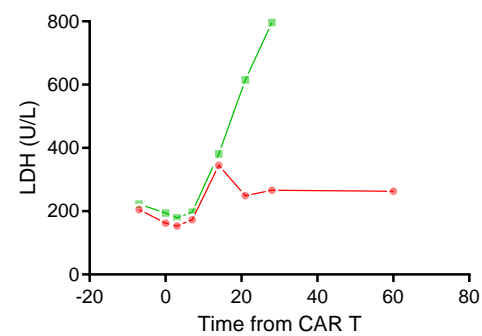**i**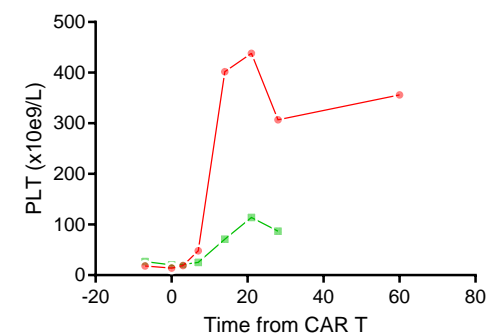

--- Pt 1    --- Pt 2

Supplement: Supplementary file 3 [file DataSheet2.pdf]
